# Development of Elite BPH-Resistant Wide-Spectrum Restorer Lines for Three and Two Line Hybrid Rice

**DOI:** 10.3389/fpls.2017.00986

**Published:** 2017-06-07

**Authors:** Fengfeng Fan, Nengwu Li, Yunping Chen, Xingdan Liu, Heng Sun, Jie Wang, Guangcun He, Yingguo Zhu, Shaoqing Li

**Affiliations:** ^1^State Key Laboratory of Hybrid Rice, Key Laboratory for Research and Utilization of Heterosis in Indica Rice of Ministry of Agriculture, Engineering Research Center for Plant Biotechnology and Germplasm Utilization of Ministry of Education, College of Life Science, Wuhan UniversityWuhan, China; ^2^College of Agronomy, Hunan Agricultural UniversityChangsha, China

**Keywords:** brown planthopper, fertility restorers, hybrid rice, cytoplasmic male sterility, photo-thermo-sensitive genic male sterility, molecular marker selection

## Abstract

Hybrid rice has contributed significantly to the world food security. Breeding of elite high-yield, strong-resistant broad-spectrum restorer line is an important strategy for hybrid rice in commercial breeding programs. Here, we developed three elite brown planthopper (BPH)-resistant wide-spectrum restorer lines by pyramiding big-panicle gene *Gn8.1*, BPH-resistant genes *Bph6* and *Bph9*, fertility restorer genes *Rf3, Rf4, Rf5*, and *Rf6* through molecular marker assisted selection. Resistance analysis revealed that the newly developed restorer lines showed stronger BPH-resistance than any of the single-gene donor parent Luoyang-6 and Luoyang-9. Moreover, the three new restorer lines had broad spectrum recovery capabilities for Honglian CMS, Wild abortive CMS and two-line GMS sterile lines, and higher grain yields than that of the recurrent parent 9,311 under nature field conditions. Importantly, the hybrid crosses also showed good performance for grain yield and BPH-resistance. Thus, the development of elite BPH-resistant wide-spectrum restorer lines has a promising future for breeding of broad spectrum BPH-resistant high-yield varieties.

## Background

The world food security is a huge challenge for human. Hybrid rice has contributed significantly to the world food production in the last decades, due to its great yield advantage over the inbred lines (Weng et al., 2008). It has been proved that heterosis utilization is one of the most effective measures to ensure food security around the world. Generally, hybrid rice can be categorized into two types, two-line based on photo-thermo-sensitive genic male sterility (GMS) and three-line based on cytoplasmic male sterility (CMS). Of which, three-line hybrid rice mainly includes Honglian (HL), Baotai (BT), and Wild abortive (WA) types based on genetic characters of cytoplasmic male sterility used for commercial production (Li et al., 2007). Unlike the two-line hybrid rice, restorer lines for three-line hybrid rice have to carry fertility restorer (*Rf*) genes that are genetically specific to some CMS type and different from each other (Li et al., 2005, 2007), which greatly limit the exploitation of heterosis because of the specific restoring-maintaining relationship. Today, there are about six *Rf* genes having been identified in rice, of which *Rf3* and *Rf4* for WA-CMS (Suresh et al., 2012; Luo et al., 2013; Tang et al., 2014), *Rf5* and *Rf6* for HL-CMS (Hu et al., 2012b; Huang et al., 2015) have been cloned or finely mapped, and can be well used for molecular breeding. This will provide us the possibility to breed wide-spectrum restorer lines that can be used not only for two-line hybrid rice, but also for HL- and WA-type hybrid rice by pyramiding these four *Rf* genes together.

Apart from the strong restoring ability, an elite restorer line should also have high combining ability, large panicles, and strong resistance to diseases and insect pests. Additive effect is one of the important factors that contribute to heterosis of rice yield by the accumulation of genetic effects from multiple dominant or semi-dominant loci (Luo et al., 2009; Birchler, 2015; Dan et al., 2015). Of which, many of rice yield-related genes/QTLs should play a key role on the yield heterosis just as *OsPPR37* and *Ghd7* do in rice (Liu et al., 2015). Interestingly, *Gn8.1*, a newly identified gene from wild rice *Oryza longistaminata* was suggested conferring rice big panicle and high grain yield but little side effects on the rice development, and showed a semi-dominant inheritable model, expression of *Gn8.1* can greatly increase grain number and yield (unpublished data). Thus, *Gn8.1* has great potential in developing elite restorer lines because of its high yield potential and vast pollen for production of more F_1_ seeds. Brown planthopper (BPH) is one of the severe destructive insect pests in rice production (Zhao et al., 2016). The utilization of BPH-resistant varieties has been recognized as the most effective and environment-friendly measure against detriment from brown planthopper to rice production (Suh et al., 2011). Today, there are over 30 BPH-resistant genes having been genetically defined (Zhao et al., 2016). Of which, both *Bph6* (Qiu et al., 2010) and *Bph9* (Zhao et al., 2016), identified from cultivar Swarnalata and Pokkali respectively, are suggested conferring strong resistance to the brown planthopper, and show tremendous potentiality in developing brown planthopper-resistant rice varieties.

In order to develop an elite BPH-resistant restorer line with wide restoring ability and good combining ability, we pyramided big-panicle gene *Gn8.1*, BPH-resistant genes *Bph6* and *Bph9*, fertility restorer genes *Rf3, Rf4, Rf5*, and *Rf6* into elite rice 9,311 based on molecular marker assisted selection, and successfully developed three elite BPH-resistant wide-spectrum restorer lines. These new restorer lines not only significantly improved the resistance against brown planthopper, but also showed good performance for the production of two-line, HL- and WA-type three line hybrid rice.

## Materials and methods

### Plant materials and brown planthopper population

9,311, a BPH-susceptible elite restorer line for HL-CMS and two-line hybrids, was used as the recurrent parent. L1880, a chromosome segment substitution line (CSSL) carrying *Gn8.1* gene in 9,311 genetic background, was used as the donor parent for large panicle. Shuhui527, an elite restorer line for WA-CMS and two-line hybrids, was used as the donor parent for Wild-Abortive type restorer gene. Luoyang-6 and Luoyang-9, CSSLs carrying *Bph6* and *Bph9* in 9,311 genetic background, respectively, were used as the donor parents for BPH-resistance. In addition, seven different types of CMS or GMS sterile lines were used as test parents in this study (Table S1). The brown planthopper populations used for infestation were collected from rice experimental field in Wuhan University.

### Evaluation for plant resistance against brown planthopper

A seedling bulk test was conducted to evaluate the brown planthopper resistance of rice populations following previously described methods (Huang et al., 2001). The seeds of each rice were pre-germinated to ensure that all seedlings were grown consistently. Detailedly, about 20 seeds were sown in a 9 cm-diameter plastic cup with three replications. At the third-leaf stage, the seedlings were thinned to 10 plants per cup and infested with second to third-instar nymphs of brown planthopper with eight insects per seedling. When all of the seedlings of susceptible control died, the plants of other rice were examined and each seedling was given a score of 1 to 9 according to the method of Huang et al. (2001).

### Foreground selection and background profiling by SSR marker analysis

Rice genomic DNA for PCR analysis was extracted from young leaves using the CTAB method (Murray and Thompson, 1980). Seven gene-specific PCR markers, Indel33, RM16994, RM28438, RM10318, RM6100, RM25661, and Indel200-1, tightly linked to the target genes *Gn8.1, Bph6, Bph9, Rf3, Rf4, Rf5*, and *Rf6*, respectively, were used in foreground selection of the target genes in each generation of intercross and backcross (Table S2). PCR was performed in a total volume of 10 μl containing 50 ng of DNA template, 1 μl 10 × PCR buffer, 0.25 μM of each primer, 75 μM of each dNTP, and 0.25 units of Taq DNA polymerase (Fermentas). The PCR amplification condition was as follows: one cycle at 94°C for 5 min, followed by 36 cycles of 94°C for 30 s, 50~60°C (dependent on the primers) for 30 s, 72°C for 45 s, and a final incubation at 72°C for 5 min. All the PCR products were analyzed by electrophoresis on agarose gel.

A total of 672 SSR markers distributed evenly on the 12 chromosomes with an average marker interval of 553.4 kb were used for rice genetic polymorphism survey, and 225 SSR markers were found to be polymorphic between the recurrent and donor parents (Table S3). Then, the 225 markers were used to identify the background of the pyramided lines. The physical map was drawn using MapMaker Version 3.0 (Lander et al., 1987). The percentages of chromosome segments from recurrent parent in pyramided lines were reckoned following the graphical genotyping procedure (Xi et al., 2006; Suh et al., 2013).

### Evaluation of agronomic traits in the field

All of the rice lines were planted in the experimental field of Ezhou in summer, and Hainan in winter during 2011–2016. Among them, the hybrid combinations and their parents used for evaluating the restoring ability and production potential of the new restorer line were planted in the Wuhan University Ezhou Experimental Base in the summer of 2016. Seedlings were transplanted in a five-row plot with 12 plants per row by 20 × 26 cm spacing under a randomized complete block design with three replications. Five representative plants in the middle of each repeat were used to measure growth duration (GD), plant height (PH), panicle number (PN), number of grain (NG), number of grain per panicle (NGP), spikelet fertility (SF), 1,000-grain weight (GW), and yield per plant (YP). The statistical analyses were performed with SPSS Statistics 20 (IBM, USA).

## Results

### Development of BPH-resistant wide-spectrum restorer lines by pyramiding

**Figure 1:**
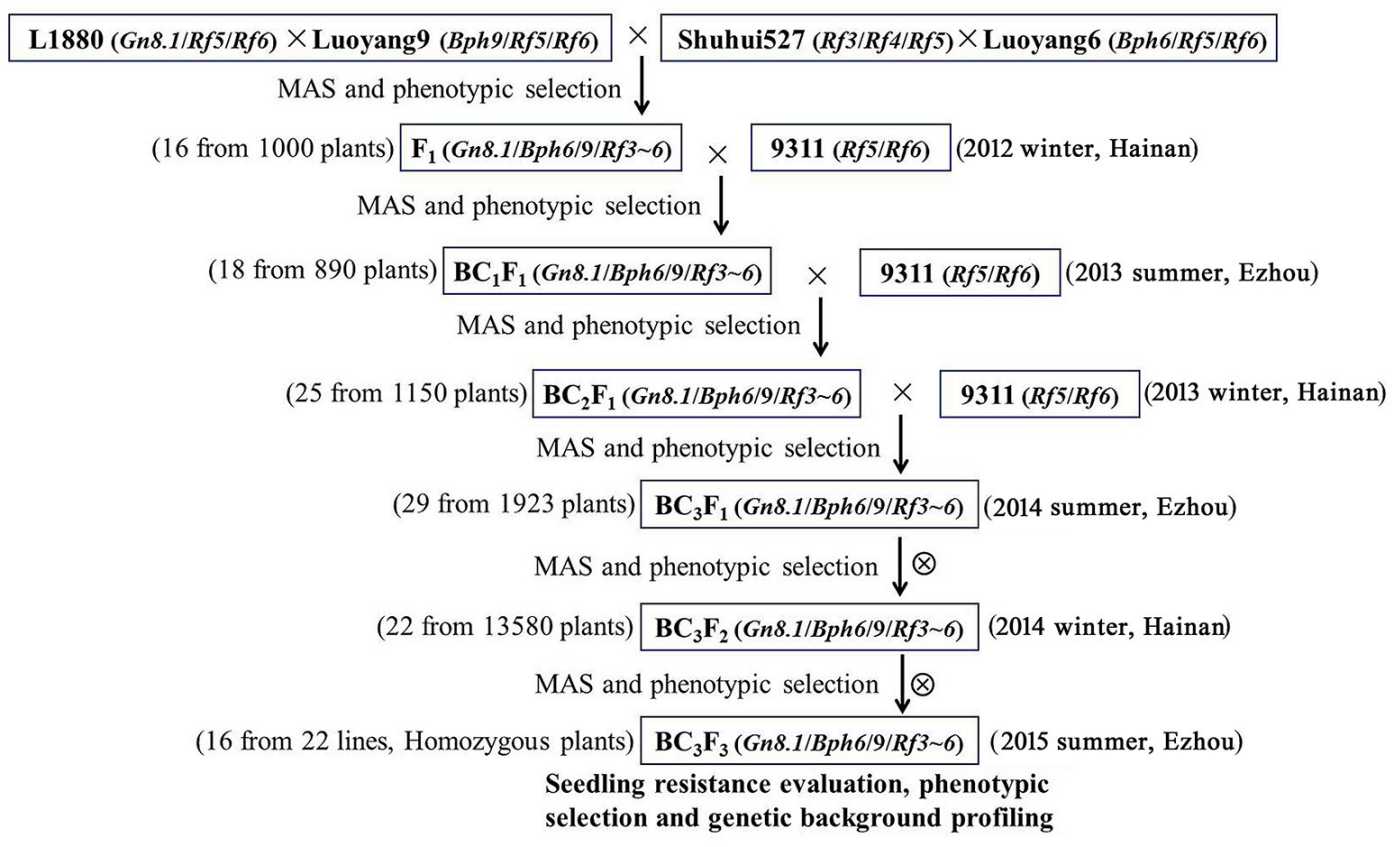
Schematic flow for the development of BPH-resistant wide-spectrum restorer lines.

As shown in the schematic flow (Figure 1) of the development of the BPH-resistant wide-spectrum restorer lines, five parents including L1880, Luoyang-6, Luoyang-9, and typical restorer line Shuhui527 and 9,311 were used in this breeding program. Of which, L1880 derived from crosses of 9,311 × *O. longistaminata* carries big panicle gene *Gn8.1*, Luoyang-6 and Luoyang-9 carry *Bph6* and *Bph9*, respectively, Shuhui527 carries restoring gene *Rf3* and *Rf4*, and 9,311 carries restoring gene *Rf5* and *Rf6*. During the breeding process, foreground selection was performed from generation F_1_ to BC_3_F_3_ to screen the plants having the desirable alleles of the seven target genes using molecular markers (Table S2). Only progenies carrying all of target alleles were selected for backcross or selfing (Figures S1–S3). Meanwhile, phenotype identification was also conducted so as to eliminate plants with linkage drag traits (Figures S4, S5; Tables S4, S5). In detail, 20 positive F_1_ plants with seven target genes were identified and genotyped using the target gene markers from a ~1,000 F_1_ population derived from intercross of L1880/Luoyang-9//Shuhui527/Luoyang-6. From which, 16 plants were identified having all of the seven target genes (*Gn8.1/Bph6/9/Rf3*~*6*), and further backcrossed as maternal parents with 9,311. In the BC_1_ generation, 18 out of 890 plants were identified as our expected candidates after careful genotyping and phenotyping. Similarly, the selected BC_1_, BC_2_ plants were then backcrossed with 9,311 so as to eliminate the unexpected background. Then, 29 BC_3_F_1_ plants were selected for selfing until genetically stable in BC_3_F_3_. Finally, 22 stable BC_3_F_3_ lines with target genes were developed from 13,580 plants. In consideration of the integrative agronomic traits and the presence of homozygous marker alleles for the seven target genes, 16 lines were selected as potential candidates from the 22 BC_3_F_3_ progenies (Figure S3; Table S5).

### Evaluation of brown planthopper-resistance and agronomic traits of pyramided lines

**Figure 2:**
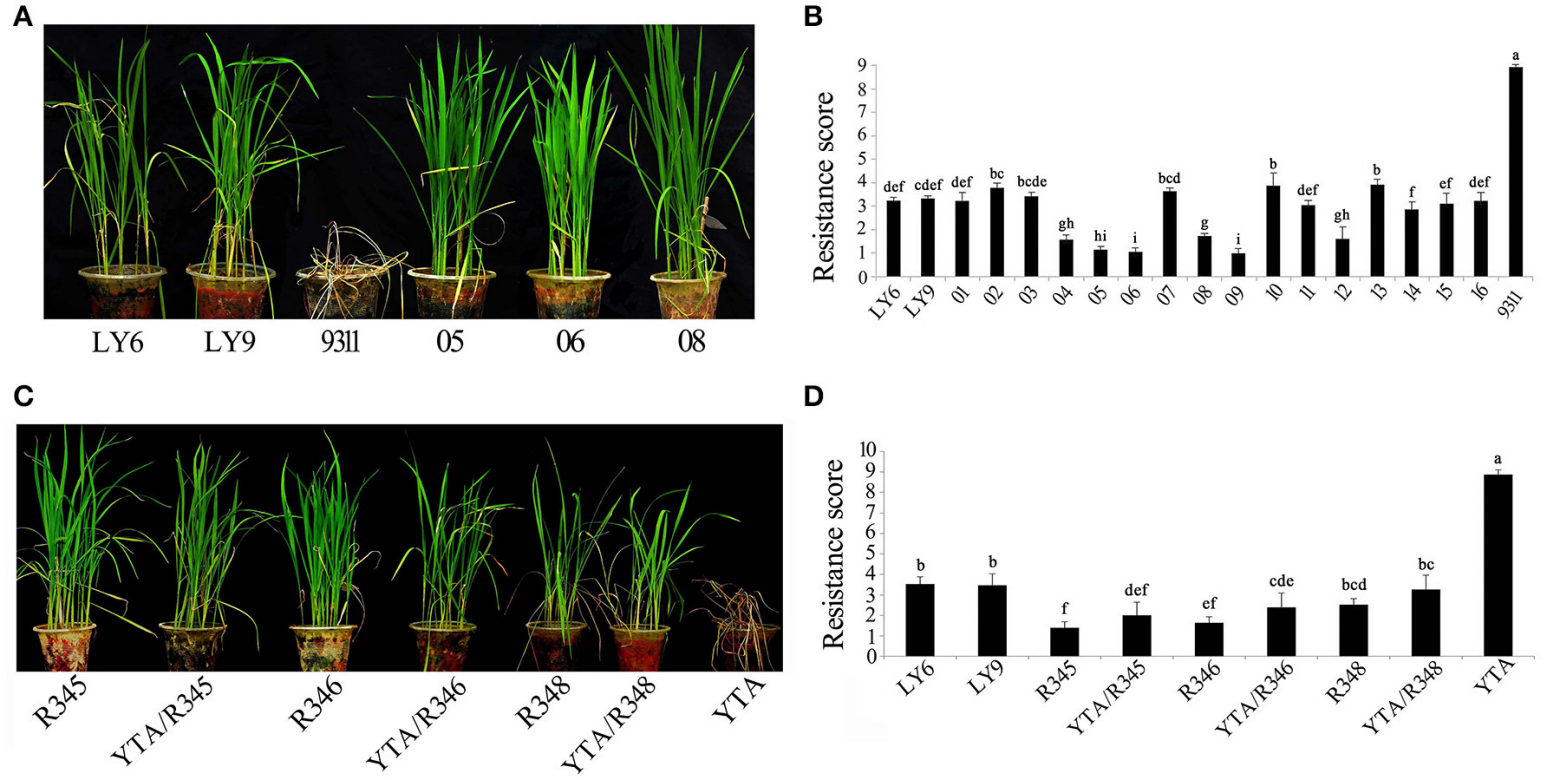
BPH-resistance test of the BPH-resistant wide-spectrum restorer lines at the seedling stage. **(A,B)**: BPH-resistance phenotype **(A)** and scores **(B)** of the pyramided restorer lines. LY6 and LY9, Resistant control line Luoyang-6 and Luoyang-9, respectively (The same as below); 9,311, susceptible control; 01~16: BC_3_F_3_ lines. **(C,D)**: BPH-resistance test **(C)** and scores **(D)** of the restorer lines and hybrid rice F_1_s. YTA, Susceptible control Yuetai A. Letters indicate a significant difference at the 5% significance level by the least significant difference test.

To test whether the selected pyramiding lines can improve the BPH-resistance, we evaluated their resistance to brown planthopper at seedling stage under greenhouse conditions, using Luoyang-6 and Luoyang-9 (donor parents) as resistant control, and 9,311 (recurrent parent) as a susceptible control. After inoculated for 1 week, when all the 9,311 seedlings were dead, the pyramided lines were still green (Figure 2A). Resistance scoring showed that BPH-resistance indexes of the part of pyramided lines ranged from 1.0 to 1.8, significantly less than that of the Luoyang-6 (3.2) and Luoyang-9 (3.3) (Figure 2B). This result implied that these pyramided lines' BPH-resistance were significantly stronger than that of the single-gene donor parents Luoyang-6 and Luoyang-9, although both of them showed high resistant level.

**Figure 3:**
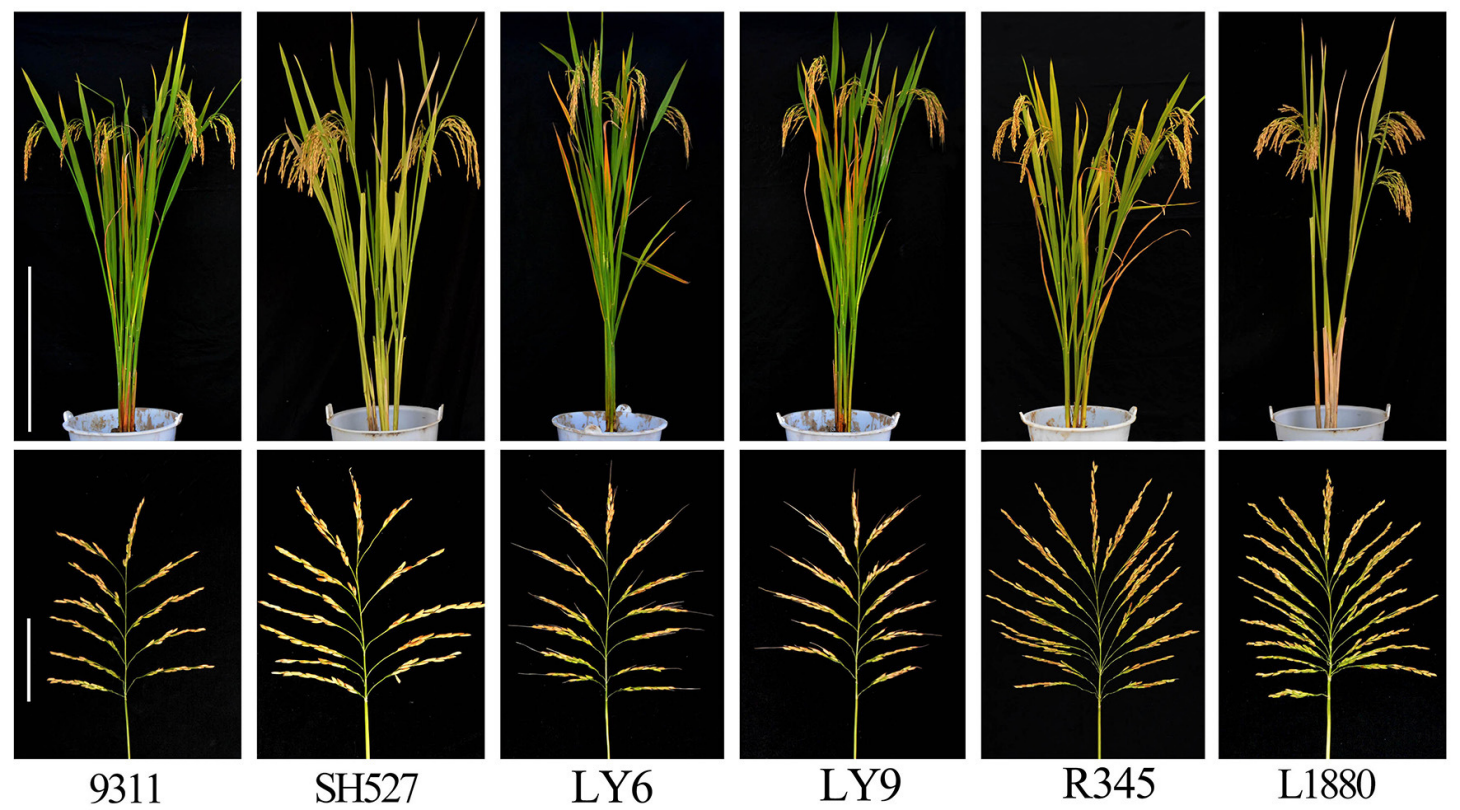
Gross plant morphology of the parent lines and newly developed BPH-resistant wide-spectrum restorer line R345. Upper panel represents plant stature, scale bar = 50 cm; lower panel represents panicle, scale bar = 10 cm.

Importantly, comparing with the recurrent parent 9,311, these pyramided lines, especially line 05, 06, and 08 all showed excellent performance in the field test. The traits such as growth period, plant height, panicle number and 1,000-grain weight of the three pyramided lines were almost the same as that of 9,311. However, the panicle size and grain number of 05, 06, and 08 were significantly larger than that of 9,311. This is a desirable agronomic trait for increasing yield. As a result, even if the spikelet fertility of 05, 06, and 08 were slightly declined, their yield per plant were still increased by 77.3, 66.1, and 85.1%, respectively than that of 9,311 (Table 1). Thus, the pyramided lines 05, 06, and 08 were finally chosen as the potential elite BPH-resistant restorer lines based on their excellent performance, and renamed as R345, R346, and R348, respectively (Figure 3).

**Table 1:** Agronomic traits of the BPH-resistant wide-spectrum restorer lines and parents.

**Rice lines**	**GD**	**PH (cm)**	**PN**	**NG**	**NGP**	**SF (%)**	**GW (g)**	**YP (g)**
9311	130 ± 1.0^b^	123 ± 6^bc^	7.0 ± 1.0^c^	1193 ± 133^c^	171 ± 6^b^	92.2 ± 0.8^a^	29.2 ± 0.2^a^	32.2 ± 4.0^c^
L1880	132 ± 1.0^a^	126 ± 5^b^	7.0 ± 1.0^c^	2018 ± 333^b^	288 ± 8^a^	88.6 ± 1.3^b^	29.4 ± 0.4^a^	52.3 ± 7.4^b^
SH527	122 ± 0.6^d^	120 ± 4^c^	8.2 ± 0.3^a^	1352 ± 28^c^	169 ± 3^b^	87.3 ± 1.8^bcd^	29.3 ± 0.4^a^	34.5 ± 0.6^c^
LY6	132 ± 0.6^a^	130 ± 7^a^	7.0 ± 1.0^c^	1215 ± 121^c^	174 ± 8^b^	85.8 ± 2.9^d^	29.7 ± 0.1^a^	31.1 ± 4.2^c^
LY9	130 ± 0.6^b^	131 ± 5^a^	7.7 ± 0.6^abc^	1305 ± 102^c^	170 ± 1^b^	86.4 ± 0.7^cd^	29.8 ± 0.9^a^	33.6 ± 3.7^c^
R345	130 ± 1.0^b^	127 ± 8^ab^	7.3 ± 0.6^bc^	2180 ± 249^ab^	297 ± 12^a^	88.3 ± 1.1^bc^	29.7 ± 0.7^a^	57.1 ± 5.9^ab^
R346	128 ± 1.0^c^	126 ± 2^b^	7.0 ± 0.8^c^	2028 ± 101^b^	290 ± 14^a^	89.1 ± 1.3^b^	29.6 ± 1.1^a^	53.5 ± 1.2^b^
R348	130 ± 0.6^b^	123 ± 7^bc^	8.1 ± 0.2^ab^	2320 ± 121^a^	290 ± 15^a^	88.4 ± 2.7^bc^	29.1 ± 0.7^a^	59.7 ± 1.5^a^

*GD, growth duration; PH, plant height; PN, panicle number; NG, number of grains; NGP, number of grains per panicle; SF, spikelet fertility; GW, 1,000-grain weight; YP, yield per plant; the same as below. Letters indicate a significant difference at the 5% significance level by the least significant difference test. The rice lines were planted in the Wuhan University Hainan Experimental Base in the winter of 2015*.

### Genetic background profiling of the pyramided lines

**Figure 4:**
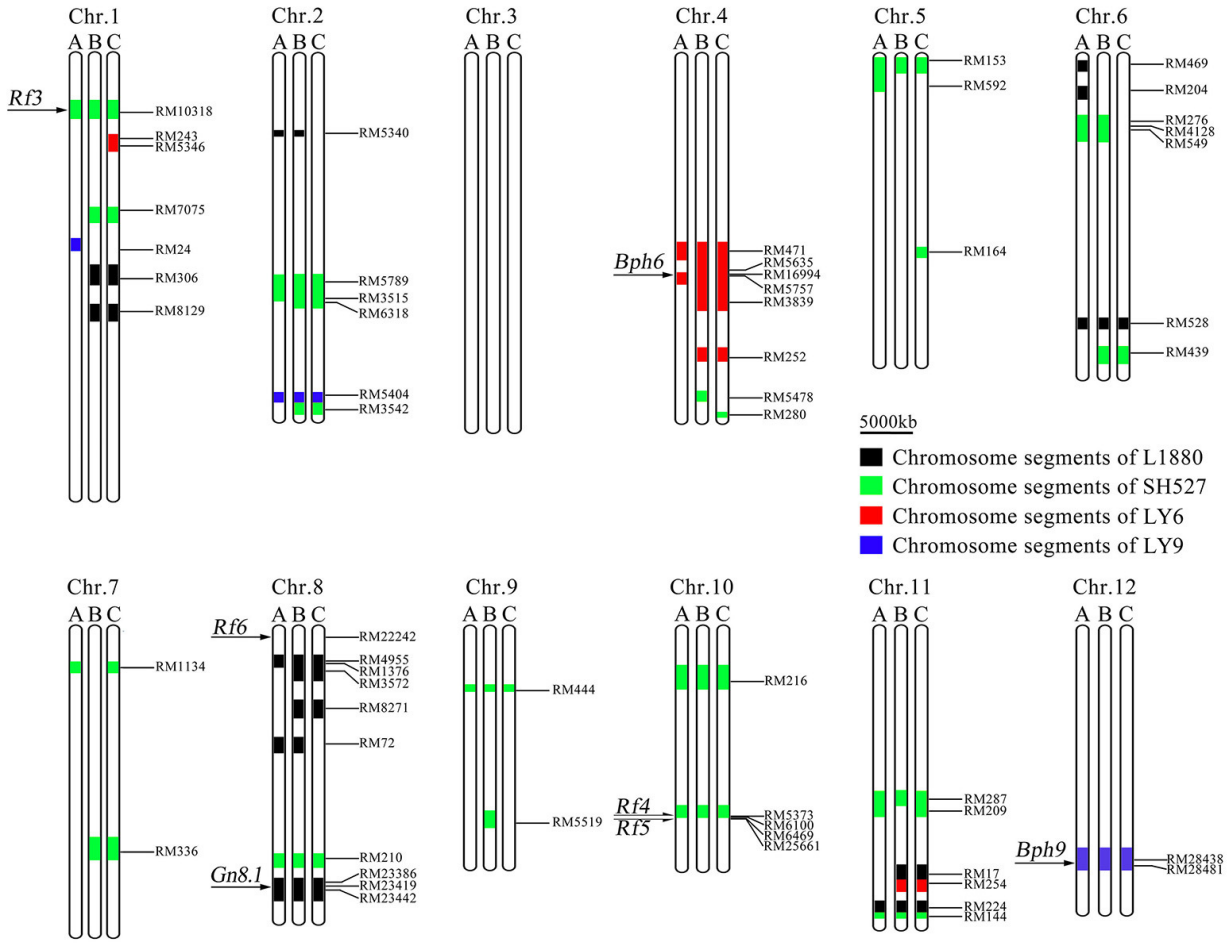
Genotypes of three newly developed BPH-resistant wide-spectrum restorer lines under 9,311 genetic background. Letters A, B and C represent line R345, R346, and R348, respectively. The black, green, red, and blue boxes indicate substituted chromosome segments of the donor parents L1880, Shuhui527, Luoyang-6, and Luoyang-9, respectively.

In order to identify the genetic background of the three pyramided lines, a total of 672 evenly distributed SSR markers were used for PCR identification, and physical maps covering the whole 12 rice chromosomes were constructed (Figure 4). The results showed that each pyramided line carried part of the donors' fragments at the seven target gene loci, even after three rounds of backcrossing with the recurrent parent 9,311. Averagely, the 9,311 genomic fragments took over 91.4, 87.5, and 88.3% in pyramided lines R345, R346, and R348, respectively (Table S3). These results demonstrated that the seven target genes *Gn8.1, Bph6, Bph9, Rf3, Rf4, Rf5*, and *Rf6* was successfully pyramided together through molecular marker selection, and R345 had the cleanest genetic background.

### Evaluation of fertility restoring ability of pyramided lines

**Figure 5:**
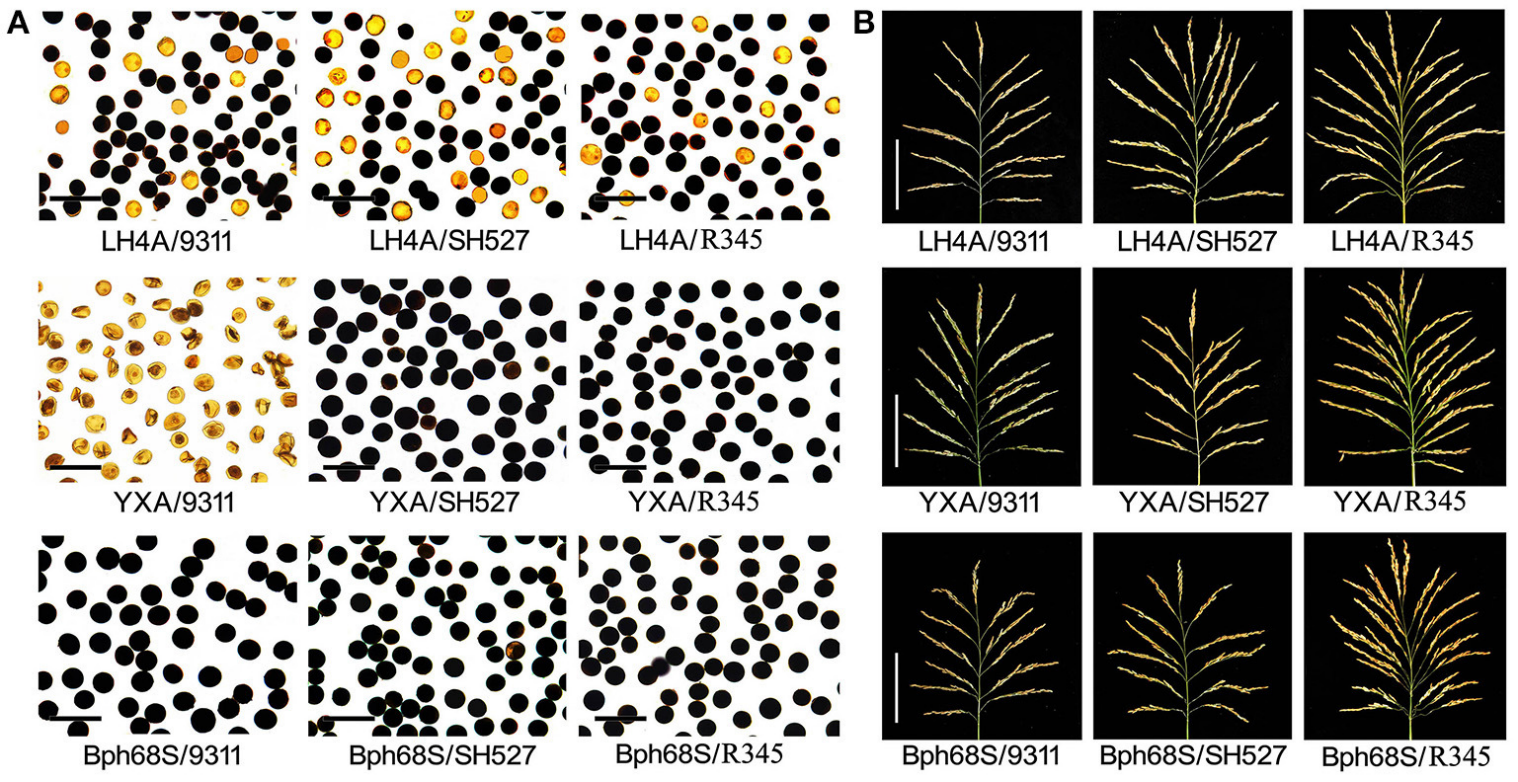
Restoring ability of the newly developed BPH-resistant wide-spectrum restorer lines in HL-CMS, WA-CMS, and two-line male sterile lines. **(A)** Pollen fertility of F_1_s assessed by 1% I_2_-KI staining. Darkly stained pollen is fertile, and slightly stained pollen is sterile, scale bar = 50 μm. LH4A, YXA and BPH68S were representative HL-CMS, WA-CMS, and two-line sterile rice, respectively. 9,311: a representative restorer line for HL-CMS and two-line GMS rice, SH527: a representative restorer line for WA-CMS and two-line GMS rice. **(B)** Mature panicles of hybrid rice F_1_s, scale bar = 10 cm.

In order to evaluate the restoring ability of the three pyramided lines, we examined the pollen and spikelet fertility of the testcrosses derived from the pyramided restorer lines. All of the six HL-type hybrid crosses (HL-CMS lines Luohong-4A and Jiahong-2A crossed with R345, R346, and R348, respectively) showed the same pollen fertility phenotype as the typical HL-type hybrid combinations Luohong-4A/9311 and Jiahong-2A/9311. Similarly, all of the WA-type and two-line hybrid combinations showed the same pollen fertility as their respective controls, and reached to almost 100% (Figure 5A; Figure S6). Moreover, we observed that the spikelet fertility showed a similar trend as that of the pollen fertility (Figure 5B; Figure S7). These results showed that the pyramided lines R345, R346, and R348 could restore all types of sterile lines, implying these elite BPH-resistant lines had wide-spectrum restoring ability.

### Performance of the testcrosses derived from the new restorer lines

To determine if the newly developed BPH-resistant wide-spectrum restorer lines can be well used in hybrid rice production, 30 hybrid rice crosses were constructed by using R345, R346, and R348, and parent restorers 9,311 (for HL-CMS and two-line) and SH527 (for WA-CMS and two-line) to testcross HL-CMS lines Luohong-4A and Jiahong-2A, WA-CMS lines Yuexiang-A and Luofei-A, and two-line sterile lines BPH68S and Guangzhan4S, respectively. Rice yields and agronomic traits including plant height, panicle number, number of grains per panicle, spikelet fertility, and 1,000-grain weight were examined when matured (Table 2; Table S6). Relative to the control of HL-type hybrid rice Luohong-4A/9311, number of grains, number of grains per panicle, and yield per plant of new HL-type combinations Luohong-4A/R345, Luohong-4A/R346, and Luohong-4A/R348 significantly increased. Similarly, for the WA-CMS, hybrid crosses derived from Yuexiang-A and restorer lines R345, R346, and R348 performed better than that of the testcrosses derived from restorer line SH527, showing significant improvements in yield per plant, although the growth duration of the combinations from Yuexiang-A crossed with the three new restorer lines were prolonged. For the two-line hybrid rice, the crosses derived from the three new restorer lines also showed significantly yield advantage over the controls. Then, we tested the resistance of the hybrid rice derived from R345, R346, and R348 and HL-CMS line Yuetai-A (BPH-susceptible), respectively. When the Yuetai-A seedlings almost completely died, Yuetai-A/R345, Yuetai-A/R346, and Yuetai-A/R348 showed normal or only slightly endangered symptoms (Figure 2C), and even a higher resistant level than the resistant controls (Figure 2D). These results indicated that the hybrid rice derived from the new restorer lines not only had strong resistance to the brown planthopper, but also high yield potential.

**Table 2:** Agronomic traits of the hybrid rice combinations derived from BPH-resistant wide-spectrum restorer lines and commercial sterile lines.

**Combinations**	**GD**	**PH (cm)**	**PN**	**NG**	**NGP**	**SF (%)**	**GW (g)**	**YP (g)**
LH4A/9311	128 ± 1.0^cd^	125 ± 4^bc^	7.3 ± 0.6^cd^	1543 ± 74^ghi^	211 ± 14^de^	74.6 ± 2.5^d^	25.7 ± 0.9^bcde^	29.6 ± 1.0^fg^
LH4A/SH527	127 ± 2.0^d^	120 ± 4^de^	9.7 ± 0.6^a^	2120 ± 258^bcde^	219 ± 18^d^	49.2 ± 3.6^e^	24.7 ± 0.5^fg^	25.6 ± 1.0^g^
LH4A/R345	128 ± 0.6^cd^	124 ± 2^cd^	9.3 ± 0.6^ab^	2529 ± 31^a^	271 ± 14^abc^	76.2 ± 2.0^cd^	25.6 ± 0.6^def^	49.3 ± 1.1^ab^
LH4A/R346	128 ± 0.6^cd^	123 ± 2^cd^	8.0 ± 1.0^abcd^	2081 ± 203^cde^	261 ± 14^bc^	77.6 ± 2.7^bcd^	25.1 ± 0.6^efg^	40.4 ± 1.8^cd^
LH4A/R348	127 ± 1.0^d^	125 ± 2^c^	9.3 ± 1.2^ab^	2518 ± 321^ab^	270 ± 15^abc^	76.6 ± 5.1^bcd^	25.3 ± 0.7^ef^	48.4 ± 1.9^ab^
YXA/9311	121 ± 1.2^f^	120 ± 2^de^	7.7 ± 0.6^bcd^	1929 ± 112^defg^	252 ± 10^c^	0^f^	–	–
YXA/SH527	114 ± 0.6^g^	117 ± 3^e^	8.7 ± 1.5^abcd^	1639 ± 215^fghi^	190 ± 9^ef^	81.0 ± 3.3^ab^	25.7 ± 0.4^bcde^	34.0 ± 3.0^ef^
YXA/R345	123 ± 0.6^e^	117 ± 2^e^	8.0 ± 1.0^abcd^	2295 ± 153^abcd^	288 ± 19^a^	83.6 ± 3.7^a^	27.0 ± 0.5^a^	51.7 ± 0.6^a^
YXA/R346	122 ± 1.0^ef^	117 ± 2^e^	7.7 ± 1.2^bcd^	2129 ± 290^abcde^	278 ± 4^ab^	80.7 ± 3.1^abc^	25.6 ± 0.2^cde^	43.9 ± 4.5^bc^
YXA/R348	121 ± 1.2^f^	116 ± 4^e^	7.3 ± 0.6^cd^	1999 ± 136^def^	273 ± 19^abc^	82.9 ± 1.6^a^	24.2 ± 0.6^g^	40.1 ± 2.6^cd^
Bph68S/9311	136 ± 0.7^a^	131 ± 2^a^	7.7 ± 1.5^bcd^	1385 ± 131^i^	183 ± 21^f^	77.3 ± 1.9^bcd^	26.8 ± 0.6^a^	28.7 ± 3.1^fg^
Bph68S/SH527	129 ± 1.5^bc^	129 ± 5^ab^	8.7 ± 1.2^abcd^	1510 ± 145^hi^	175 ± 12^f^	75.2 ± 2.9^d^	26.5 ± 0.4^abc^	30.1 ± 2.7^fg^
Bph68S/R345	131 ± 1.2^b^	132 ± 3^a^	9.0 ± 1.7^abc^	2462 ± 555^abc^	272 ± 11^abc^	73.9 ± 2.8^d^	26.5 ± 0.6^abcd^	47.9 ± 9.5^ab^
Bph68S/R346	128 ± 0.6^cd^	131 ± 3^a^	7.3 ± 0.6^cd^	1851 ± 216^efgh^	252 ± 11^c^	74.7 ± 1.7^d^	26.7 ± 0.5^a^	36.8 ± 3.1^de^
Bph68S/R348	123 ± 1.5^e^	132 ± 3^a^	7.0 ± 1.7^d^	1830 ± 270^efgh^	265 ± 26^abc^	76.2 ± 3.0^bcd^	26.5 ± 0.3^ab^	36.8 ± 3.6^de^

*GD, growth duration; PH, plant height; PN, panicle number; NG, number of grains; NGP, number of grains per panicle; SF, spikelet fertility; GW, 1000-grain weight; YP, yield per plant. LH4A, Honglian-CMS line Luohong-4A; YXA, WA-CMS line Yuexiang-A; Bph68S, a two-line photo-thermo-sensitive genic male sterile line. Letters indicated a significant difference at the 5% significance level by the least significant difference test. “–” indicated no investigation results. The combinations were planted in the Wuhan University Ezhou Experimental Base in the summer of 2016*.

## Discussion

Sustainable development of hybrid rice depended greatly on the construction of elite CMS/GMS lines and restorers. Pyramiding multiple favorable traits in a commercial restorer is considered to be one of the most effective approaches to create elite restorer lines. However, it is often difficult to reach such a goal using traditional breeding methods because of the inefficient selection and unexpected linkage drags. Thus, the molecular assisted selection becomes an important strategy in rice breeding programs for the advantage of rapid recovery of the background of the recurrent parents (Suh et al., 2013; Fan et al., 2015; Shamsudin et al., 2016). In this study, seven favorable genes were pyramided into 9,311 by using tightly linked molecular markers within six generations. For the reason, apart from the use of CSSLs with the genetic background of recurrent parent 9,311 which can accelerate to purify the genetic background, phenotypic selection that is combined with genotyping with the molecular markers that are tightly linked to the target genes can quickly reduce the linkage drag which may show unexpected side effects on the plant performance. Thus, the newly developed restorer lines showed good performance in the two-line and three-line hybrid rice production for their desirable characters including BPH-resistance, wide restoring ability, and high yield potentiality.

It is well-known that HL- and WA-CMS can be restored by *Rf3*/*Rf4* and *Rf5*/*Rf6* gene pairs, respectively (Luo et al., 2013; Huang et al., 2015). Different fertility restoring patterns limit exploitation of the heterosis potential of the three-line hybrid rice. If pyramiding of these *Rf* genes together to construct a wide-spectrum restorer line will break the isolation between different CMS types. Although gene pyramiding has been successfully used to improve rice grain yield, quality, and resistance in the last decades (Hittalmani et al., 2000; Zhou et al., 2003; Kumar et al., 2016), no reports are openly available for breeding wide-spectrum restorers by pyramiding of multiple *Rf* genes. In this study, the restorer lines carrying *Rf3, Rf4, Rf5*, and *Rf6* showed good fertility restoration for WA- and HL- three-line hybrid rice, and also two-line hybrid rice (Figure 5). It implies that these four *Rf* genes are functionally compatible each other, and these wide-spectrum restorers can be effectively used for breeding of different type hybrid rice.

Apart from high fertility restoring ability, strong resistance of the parents to diseases and insect pests are also important requirements for sustainable development of hybrid rice. As for brown planthopper, it is well recognized that the most efficient way to conquer this pest is to breed BPH-resistant rice by pyramiding multiple functional genes against brown planthopper. It is reported that rice carried *Bph3* and *Bph27(t)* had higher resistance than *Bph3* or *Bph27(t)* introgression lines (Liu et al., 2016). Rice pyramided *Bph14, Bph15*, and *Bph18* genes, similarly, showed higher resistance than those two genes pyramided lines, and the two genes pyramided lines also higher than single gene lines (Hu et al., 2012a, 2013). In this study, *Bph6* and *Bph9* were selected as target genes against brown planthopper, due to their advantages in BPH-resistance. Of which, *Bph9* shows durable and broad resistance to brown planthopper biotypes 1, 2, and 3 (Zhao et al., 2016). *Bph6* is also a high BPH-resistant gene, especially against brown planthopper biotype 4 (Qiu et al., 2010). As observed in the test, the restorer lines with both *Bph6* and *Bph9* and even their hybrid rice all showed higher resistance than any of the single-gene donor parents (Figure 2). It means that *Bph6* and *Bph9* together can confer rice broad, higher and durable resistance to all four brown planthopper biotypes, and these two genes had great potential in future hybrid rice breeding programs.

## Conclusions

We developed three elite BPH-resistant wide-spectrum restorer lines by multiple genes pyramiding through MAS. These new restorer lines significantly improved the resistance against BPH, and showed good performance in the two-line and three-line hybrid rice production. Therefore, it can be concluded that the development of elite BPH-resistant wide-spectrum restorer lines had a promising future for breeding of broad spectrum BPH-resistant high-yield varieties.

## Author contributions

SL, YZ, GH, and FF designed the research; FF, NL, YC, XL, HS, and JW performed molecular and field experiments; FF and SL analyzed data and wrote the manuscript. All authors read and approved the final manuscript.

### Conflict of interest statement

The authors declare that the research was conducted in the absence of any commercial or financial relationships that could be construed as a potential conflict of interest.

## Supplementary material

Figure S1 PCR analysis of the parental lines and F_1_ plants. M, DNA marker DL2000, the same as below. **(A)** Indel33 for *Gn8.1*. **(B)** RM16994 for *Bph6*. **(C)** RM28438 for *Bph 9*. **(D)** RM10318 for *Rf3*. **(E)** RM6100 for *Rf4*. **(F)** RM25661 for *Rf5*. **(G)** Indel200-1 for *Rf6*.

Figure S2 PCR analysis of the parental lines and BC_3_F_2_ plants. **(A)** Indel33 for *Gn8.1*. **(B)** RM16994 for *Bph 6*. **(C)** RM28438 for *Bph 9*. **(D)** RM10318 for *Rf3*. **(E)** RM6100 for *Rf4*. **(F)** RM25661 for *Rf5*. **(G)** Indel200-1 for *Rf6*.

Figure S3 PCR analysis of the parental and BC_3_F_3_ lines. **(A)** Indel33 for *Gn8.1*. **(B)** RM16994 for *Bph6*. **(C)** RM28438 for *Bph9*. **(D)** RM10318 for *Rf3*. **(E)** RM6100 for *Rf4*. **(F)** RM25661 for *Rf5*. **(G)** Indel200-1 for *Rf6*.

Figure S4 Gross plant **(Upper panel)** and panicle **(Lower panel)** morphologies of rice. Scale bars represent 50 and 10 cm, respectively.

Figure S5 Gross plant **(Upper panel)** and panicle **(Lower panel)** morphologies of rice. Scale bars represent 50 and 10 cm, respectively.

Figure S6 Pollen fertility of hybrid combinations. **(A)** HL-type hybrid combinations. **(B)** WA-type hybrid combinations. **(C)** two-line hybrid combinations.

Figure S7 Spikelet fertility of hybrid combinations. **(A)** HL-type hybrid combinations. **(B)** WA-type hybrid combinations. **(C)** two-line hybrid combinations.

Table S1 Rice materials used in this study.

Table S2 Markers used for foreground selection of seven target genes in this study.

Table S3 Genotyping of the newly developed BPH-resistant wide-spectrum restorer lines.

Table S4 Agronomic traits performance of the parental lines and hybrid plants.

Table S5 Agronomic traits performance of the parents and BC_3_F_3_ lines.

Table S6 Agronomic traits of F_1_s.
