# Supplementary figures and images for: Development of Elite BPH-Resistant Wide-Spectrum Restorer Lines for Three and Two Line Hybrid Rice
Source: Front Plant Sci. 2017 Jun 7;8:986. doi: 10.3389/fpls.2017.00986 (PMC5461369; doi:10.3389/fpls.2017.00986)

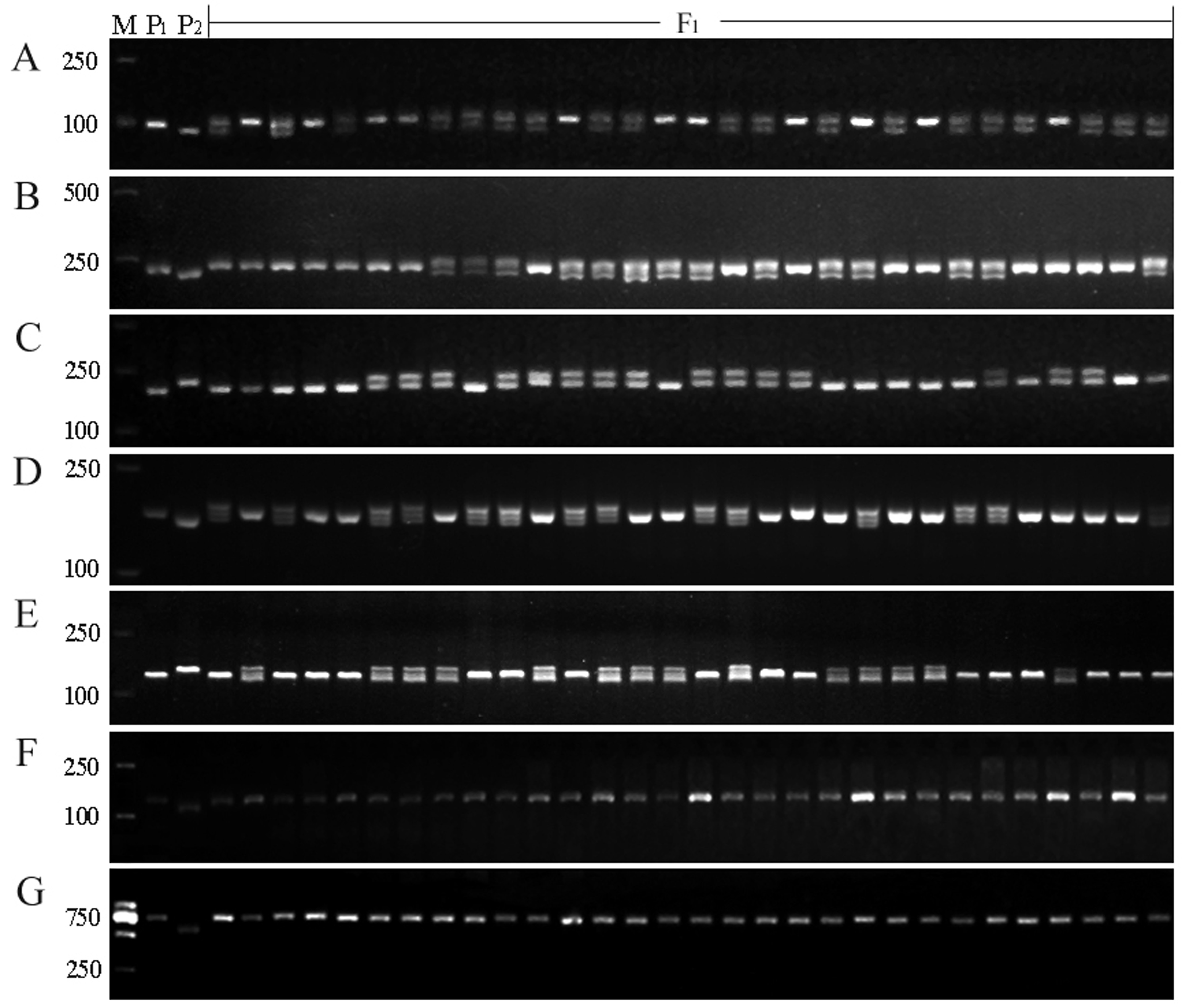

Supplement: Figure S1 — PCR analysis of the parental lines and F1 plants. M, DNA marker DL2000, the same as below. (A) Indel33 for Gn8.1. (B) RM16994 for Bph6. (C) RM28438 for Bph 9. (D) RM10318 for Rf3. (E) RM6100 for Rf4. (F) RM25661 for Rf5. (G) Indel200-1 for Rf6. [file Image1.JPEG]

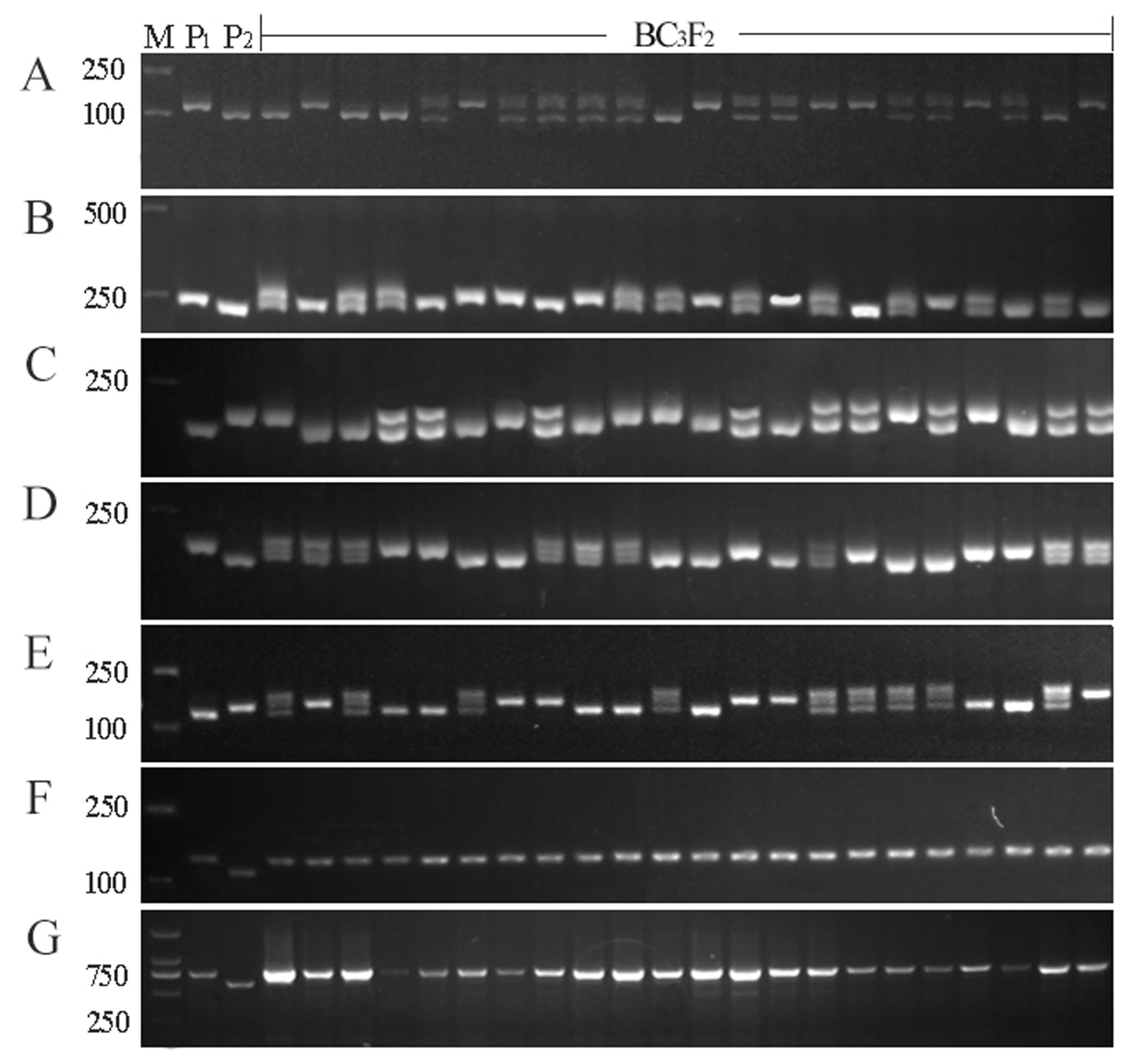

Supplement: Figure S2 — PCR analysis of the parental lines and BC3F2 plants. (A) Indel33 for Gn8.1. (B) RM16994 for Bph 6. (C) RM28438 for Bph 9. (D) RM10318 for Rf3. (E) RM6100 for Rf4. (F) RM25661 for Rf5. (G) Indel200-1 for Rf6. [file Image2.JPEG]

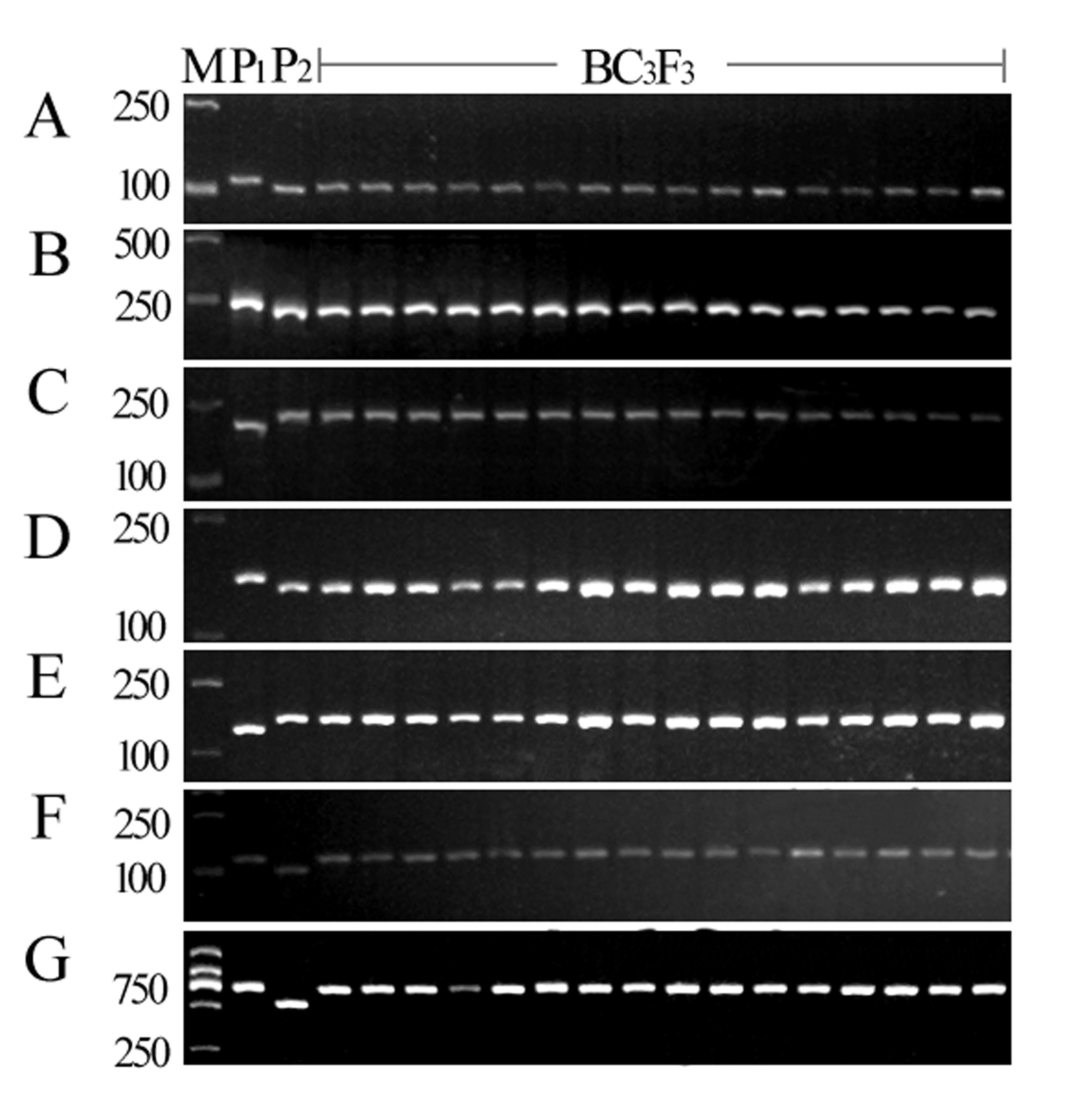

Supplement: Figure S3 — PCR analysis of the parental and BC3F3 lines. (A) Indel33 for Gn8.1. (B) RM16994 for Bph6. (C) RM28438 for Bph9. (D) RM10318 for Rf3. (E) RM6100 for Rf4. (F) RM25661 for Rf5. (G) Indel200-1 for Rf6. [file Image3.JPEG]

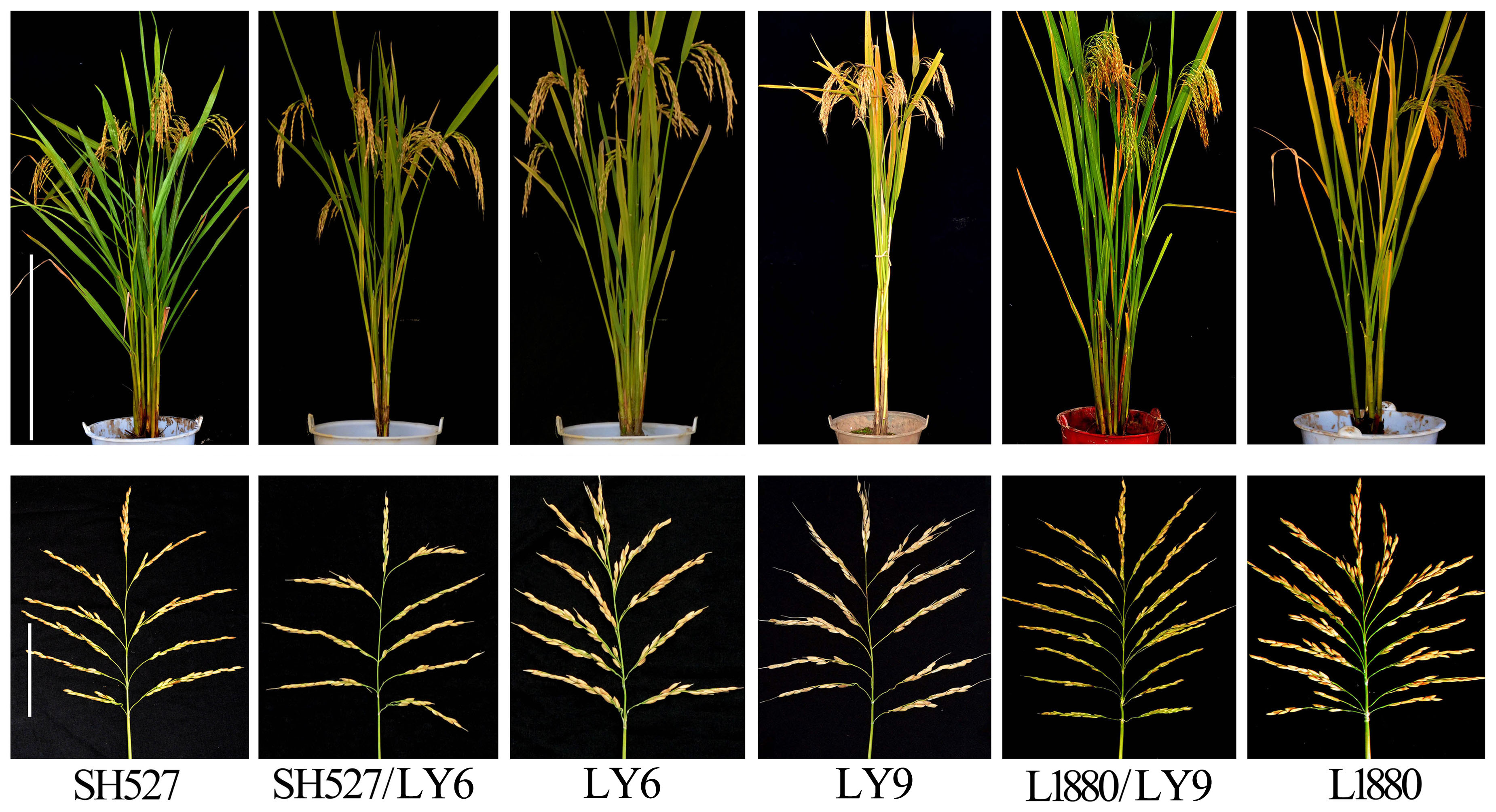

Supplement: Figure S4 — Gross plant (Upper panel) and panicle (Lower panel) morphologies of rice. Scale bars represent 50 and 10 cm, respectively. [file Image4.JPEG]

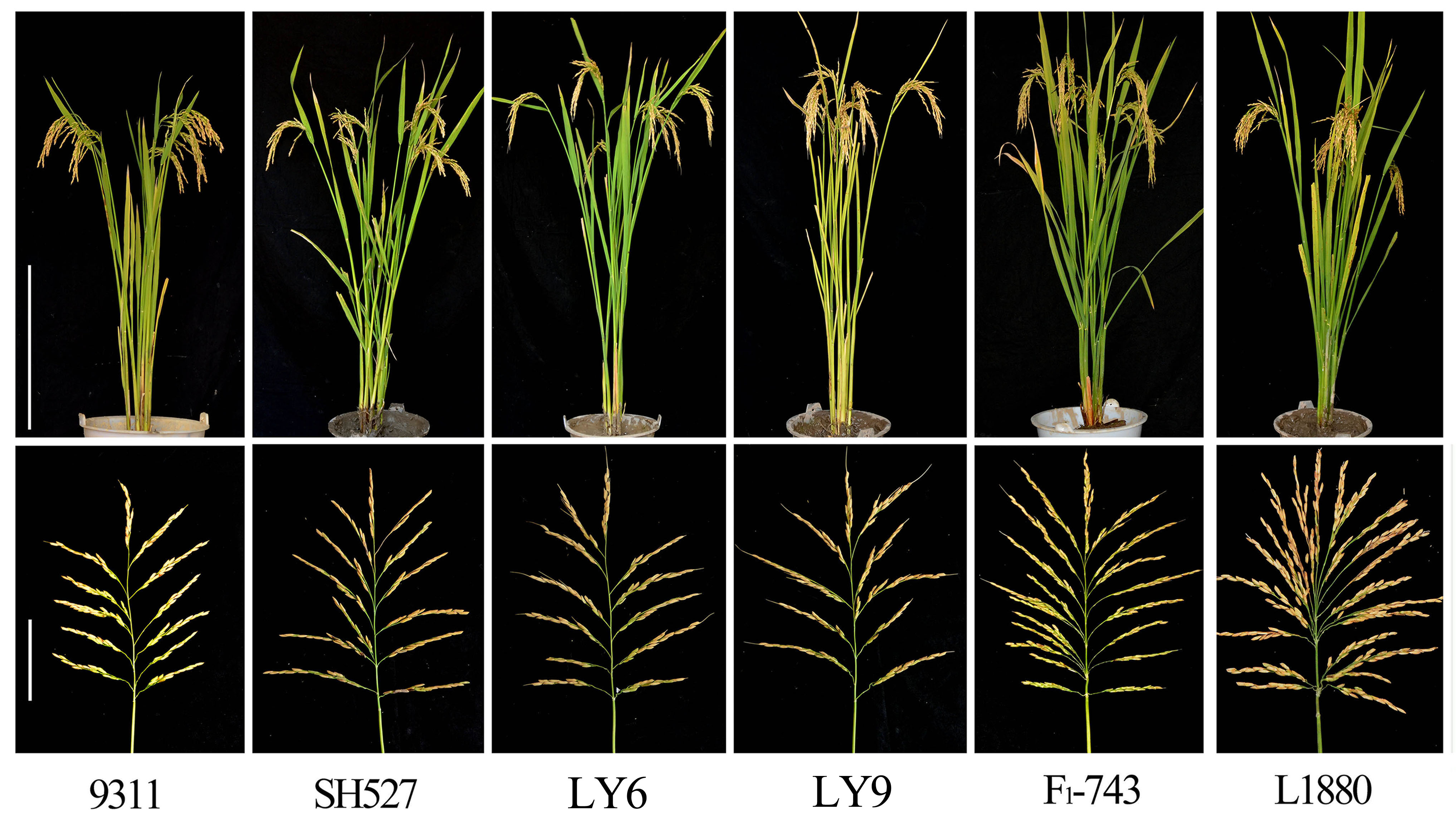

Supplement: Figure S5 — Gross plant (Upper panel) and panicle (Lower panel) morphologies of rice. Scale bars represent 50 and 10 cm, respectively. [file Image5.JPEG]

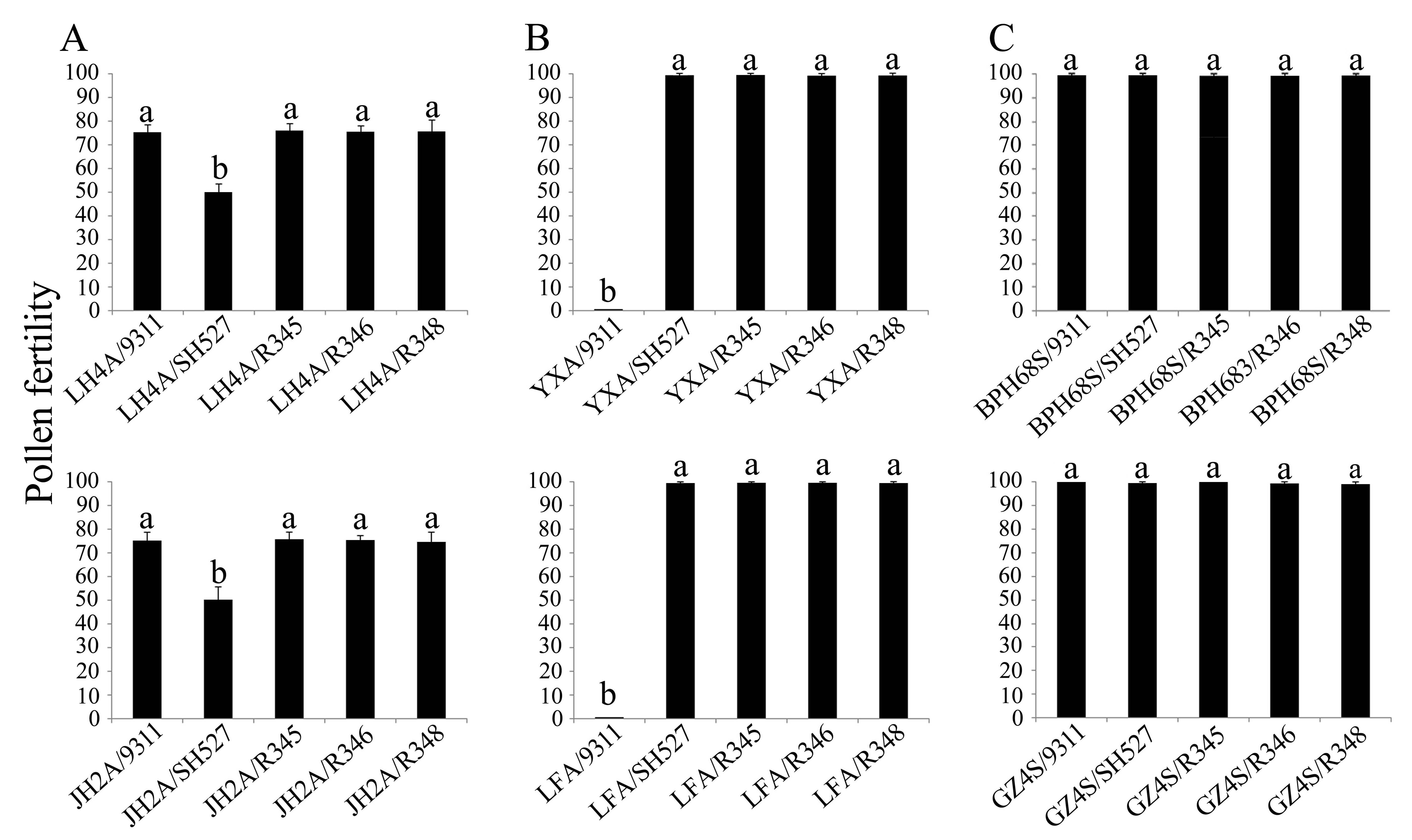

Supplement: Figure S6 — Pollen fertility of hybrid combinations. (A) HL-type hybrid combinations. (B) WA-type hybrid combinations. (C) two-line hybrid combinations. [file Image6.JPEG]

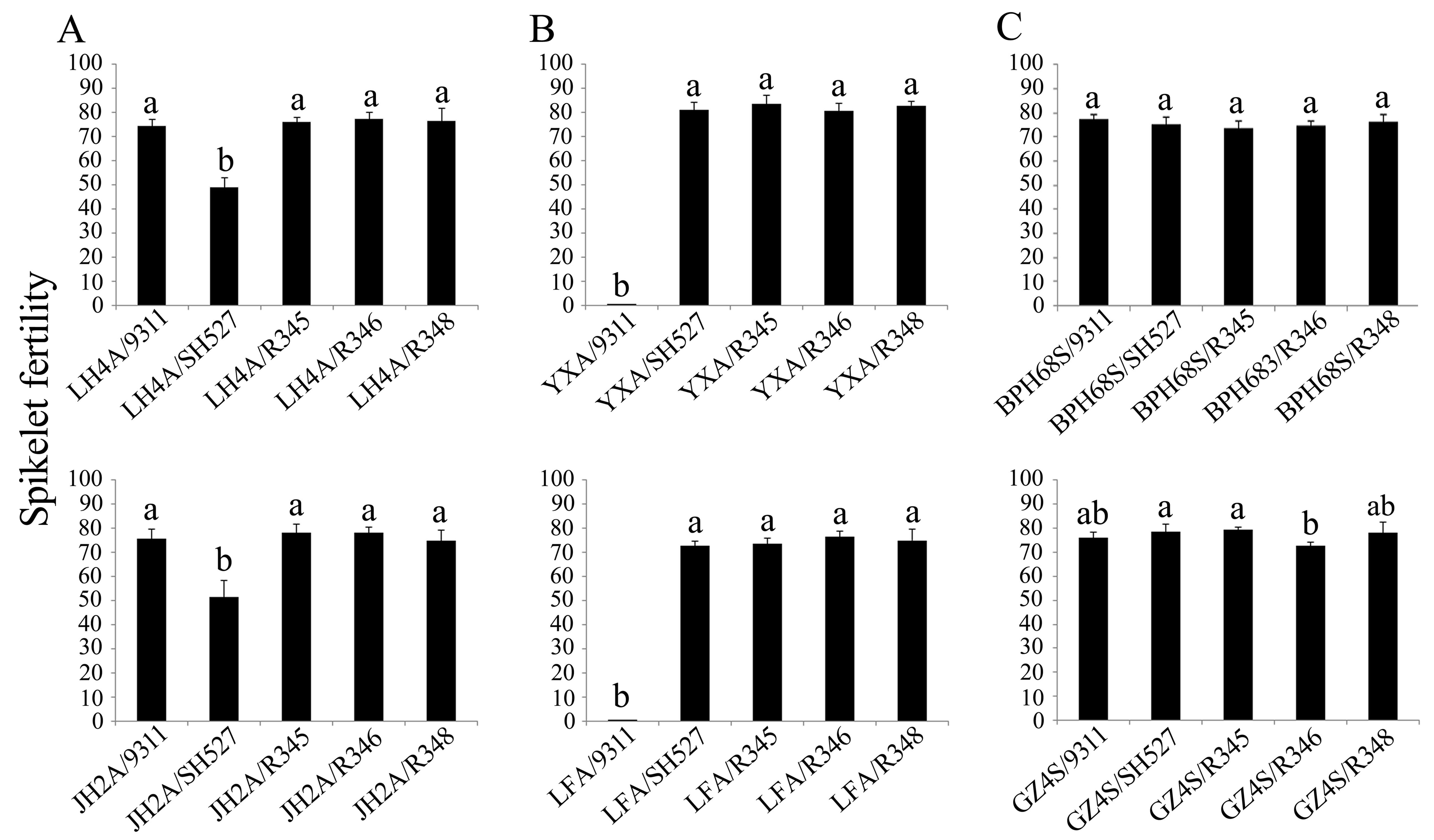

Supplement: Figure S7 — Spikelet fertility of hybrid combinations. (A) HL-type hybrid combinations. (B) WA-type hybrid combinations. (C) two-line hybrid combinations. [file Image7.JPEG]
