# Supplementary material for: Development of Elite BPH-Resistant Wide-Spectrum Restorer Lines for Three and Two Line Hybrid Rice
Source: Front Plant Sci. 2017 Jun 7;8:986. doi: 10.3389/fpls.2017.00986 (PMC5461369; doi:10.3389/fpls.2017.00986)
Supplement: Table S1 — Rice materials used in this study. [file Table1.DOCX]

**Development of elite BPH-resistant wide-spectrum restorer lines for three and two line hybrid rice**

Fengfeng Fan^1^, Nengwu Li^1^, Yunping Chen^1^, Xingdan Liu^2^, Heng Sun^1^, Jie Wang^1^, Guangcun He^1^, Yingguo Zhu^1^, Shaoqing Li^1^

^1^ State Key Laboratory of Hybrid Rice, Key Laboratory for Research and Utilization of Heterosis in Indica Rice of Ministry of Agriculture, Engineering Research Center for Plant Biotechnology and Germplasm Utilization of Ministry of Education, College of Life Science, Wuhan University, Wuhan 430072, China.

^2^ College of Agronomy, Hunan Agricultural University, Changsha 410128, China

Corresponding author:

Shaoqing Li

Email: shaoqingli@whu.edu.cn;

Tel: 86-27-68752285

Fax: 86-27-68752095

**Supplementary Tables**

**Table S1** Rice materials used in this study

| Materials | Abbreviate | Description | Gene | Remark粒重g |
| --- | --- | --- | --- | --- |
| 9311 | 9311 | Recurrent parent | *Rf5*/*Rf6* | Restorer line for HL-CMS and two-line |
| L1880 | L1880 | Donor parent | *Gn8.1*/*Rf5*/*Rf6* | CSSL-*Gn8.1* in 9311 background |
| Shuhui527 | SH527 | Donor parent | *Rf3*/*Rf4/Rf5* | Restorer line for WA-CMS and two-line |
| Luoyang-6 | LY6 | Donor parent | *Bph6/Rf5*/*Rf6* | CSSL-*Bph6* in 9311 background |
| Luoyang-9 | LY9 | Donor parent | *Bph9/Rf5*/*Rf6* | CSSL-*Bph9* in 9311 background |
| Yuetai-A | YTA | CMS line | - | HL-CMS |
| Luohong-4A | LH4A | CMS line | - | HL-CMS |
| Jiahong-2A | JH2A | CMS line | - | HL-CMS |
| Yuexiang-A | YXA | CMS line | - | WA-CMS |
| Luofei-A | LFA | CMS line | - | WA-CMS |
| BPH68S | BPH68S | GMS line | - | Two-line |
| Guangzhan4S | GZ4S | GMS line | - | Two-line |

“-” indicated these lines did not contain any of the seven genes.

**Table S2** Markers used for foreground selection of seven target genes in this study

| **Gene** | **Linked marker** | **Primer sequences** | **Reference** |
| --- | --- | --- | --- |
| ***Gn8.1*** | Indel33 | F:GCAAGAACTCGTGCCAAACAT | unpublished data |
|  |  | R:ACTGTTCTATTGCCTATTGGGGA |  |
| ***Bph6*** | RM16994 | F:TGGCAGTACACACTACAGTACATGC | Qiu et al. 2012 |
|  |  | R: AGAGGGAGGAGAGAAAGGAAGG |  |
| ***Bph9*** | RM28438 | F:GTTCGTGAGCCACAACAAATCC | Zhao et al. 2016 |
|  |  | R:GTTAAATGCTCCACCAAACACACC |  |
| ***Rf3*** | RM10318 | F:TGTCTCACACATTGCACACTTACC | Suresh et al. 2012 |
|  |  | R:GGCCTAACCCAACACATGTCC |  |
| ***Rf4*** | RM6100 | F:TCCTCTACCAGTACCGCACC | Tang et al. 2014 |
|  |  | R:GCTGGATCACAGATCATTGC |  |
| ***Rf5*** | RM25661 | F: TCTCTACCTGGCGTCCACTAGTTCG | Hu et al. 2012 |
|  |  | R: AGGTGGACGTGCTCGATCTGC |  |
| ***Rf6*** | Indel200-1 | F: CACTGCCCACCCAGATTCCTC | Huang et al. 2015 |
|  |  | R: AATCCTTGCTGCCTCATTTTGC |  |

**Table S3** Genotyping of the newly developed BPH-resistant wide-spectrum restorer lines

| Chromosomes | No. of  markers | Average interval between markers (kb) | | No. of polymorphic markers | Ration of chromosome segments from recurrent parents (%)  R345 R346 R348 | | | | |  |  |  |
| --- | --- | --- | --- | --- | --- | --- | --- | --- | --- | --- | --- | --- |
| Chrom.1 | 75 | 576.9 | 27 | | | 94.7 | 86.2 | 82.4 | |  |  |  |
| Chrom.2 | 61 | 589.1 | 20 | | | 90.0 | 85.3 | 86.6 | |  |  |  |
| Chrom.3 | 62 | 587.3 | 12 | | | 100.0 | 100.0 | 100.0 | |  |  |  |
| Chrom.4 | 62 | 572.6 | 21 | | | 93.3 | 76.9 | 78.1 | |  |  |  |
| Chrom.5 | 56 | 534.9 | 18 | | | 90.7 | 96.0 | 93.3 | |  |  |  |
| Chrom.6 | 58 | 538.7 | 26 | | | 82.7 | 84.6 | 92.3 | |  |  |  |
| Chrom.7 | 56 | 530.3 | 16 | | | 96.6 | 93.9 | 90.6 | |  |  |  |
| Chrom.8 | 54 | 526.7 | 28 | | | 80.3 | 70.5 | 76.1 | |  |  |  |
| Chrom.9 | 42 | 547.9 | 13 | | | 97.8 | 91.7 | 97.8 | |  |  |  |
| Chrom.10 | 44 | 527.4 | 16 | | | 88.8 | 88.8 | 88.8 | |  |  |  |
| Chrom.11 | 52 | 558.1 | 15 | | | 88.6 | 82.8 | 80.0 | |  |  |  |
| Chrom.12 | 50 | 550.6 | 13 | | | 93.8 | 93.8 | 93.8 | |  |  |  |
| Total(average) | 672 | 553.4 | 225 | | | 91.4 | 87.5 | 88.3 | |  |  |  |
|  |  |  | |  |  | |  | |  |  |  |  |

**Table S****4** Agronomic trait performance of the parental lines and hybrid plants

| material | PH(cm) | PN | NG | NGP数 | SF(%) | GW(g) | YP(g)粒重g |
| --- | --- | --- | --- | --- | --- | --- | --- |
| L1880 | 120±5 | 6.1±1.9 | 1809±311 | 293±15 | 89.2±2.1 | 29.4±0.3 | 46.4±5.4 |
| SH527 | 116±6 | 7.0±1.0 | 1129±193 | 165±15 | 85.6±3.9 | 30.2±0.2 | 30.2±3.6 |
| LY6 | 126±3 | 7.6±2.0 | 1238±121 | 171±35 | 81.2±3.4 | 30.1±1.7 | 28.9±3.6 |
| LY9 | 125±6 | 7.2±2.5 | 1181±162 | 168±39 | 79.5±3.7 | 31.6±0.8 | 29.3±4.7 |
| L1880/LY 9 | 127±8 | 6±2.3 | 1426±138 | 241±22 | 89.0±2.8 | 30.3±1.1 | 38.9±2.9 |
| SH527/LY 6 | 117±6 | 7.3±1.8 | 1246±283 | 168±33 | 85.2±3.0 | 29.8±1.4 | 31.1±3.1 |

Note: PH, Plant height; PN, panicle number; NG, number of grains; NGP, number of grains per panicle; SF, spikelet fertility; GW, 1000-grain weight; YP, yield per plant; the same as below.

**Table S5** Agronomic traits performance of the parents and BC_3_F_3_ lines

| material | PH(cm) | PN | NG | | NGP | SF(%) | | GW(g) | | YP(g) | |  |
| --- | --- | --- | --- | --- | --- | --- | --- | --- | --- | --- | --- | --- |
| 9311 | 117±6 | 7.7±1.2 | 1226±116 | | 161±11 | 91.9±1.5 | | 30.3±0.5 | | 34.0±3.1 | |  |
| L1880 | 118±4 | 8.0±1.0 | 2254±166 | | 283±15 | 87.5±0.5 | | 29.4±0.6 | | 58.0±3.6 | |  |
| SH527 | 115±6 | 7.7±0.6 | 1349±57 | | 176±7 | 87.1±1.9 | | 30.5±0.7 | | 35.7±1.2 | |  |
| YD6 | 122±5 | 7.7±1.2 | 1256±192 | | 164±4 | 82.5±2.3 | | 30.4±0.8 | | 31.4±4.4 | |  |
| YD9 | 124±6 | 8.2±1.5 | 1403±265 | | 168±4 | 81.7±3.4 | | 30.0±0.3 | | 34.3±5.9 | |  |
| 01 | 119±3 | 6.3±0.6 | 1715±103 | | 271±11 | 85.4±3.2 | | 29.1±0.2 | | 42.6±3.3 | |  |
| 02 | 114±6 | 6.0±1.0 | 1534±153 | | 258±18 | 84.7±1.5 | | 29.0±0.4 | | 37.7±3.1 | |  |
| 03 | 120±5 | 7.7±0.6 | 1932±101 | | 252±8 | 90.5±1.7 | | 28.6±0.3 | | 50.0±1.7 | |  |
| 04 | 117±4 | 7.3±0.6 | 1928±140 | | 263±7 | 90.3±0.7 | | 30.1±0.4 | | 52.4±5.0 | |  |
| 05 | 120±5 | 7.7±0.6 | 2343±149 | | 306±7 | 92.9±1.7 | | 29.9±0.8 | | 65.1±4.6 | |  |
| 06 | 121±7 | 8.1±1.5 | 2326±296 | | 281±16 | 89.2±2.2 | | 29.3±0.3 | | 60.6±5.9 | |  |
| 07 | 119±5 | 5.3±1.5 | 1460±390 | | 275±7 | 88.5±5.6 | | 30.3±0.4 | | 38.8±7.9 | |  |
| 08 | 115±6 | 6.8±1.2 | 1954±262 | | 294±11 | 90.9±1.8 | | 31.7±0.2 | | 56.4±7.6 | |  |
| 09 | 117±4 | 5.0±1.0 | 1481±209 | | 299±18 | 87.5±0.8 | | 29.0±0.7 | | 37.6±6.0 | |  |
| 10 | 114±7 | 6.7±1.3 | 2065±297 | | 311±10 | 84.8±1.5 | | 31.3±0.4 | | 55.0±9.5 | |  |
| 11 | 125±5 | 8.5±0.6 | 2407±115 | | 289±7 | 85.1±1.2 | | 29.0±0.8 | | 59.5±4.5 | |  |
| 12 | 115±6 | 7.3±1.2 | 1780±223 | | 244±9 | 85.4±2.8 | | 29.2±0.3 | | 44.6±7.2 | |  |
| 13 | 122±5 | 7.4±0.6 | 1993±195 | | 272±6 | 91.3±2.0 | | 28.9±0.5 | | 52.6±5.5 | |  |
| 14 | 124±7 | 5.2±0.8 | 1331±111 | | 250±7 | 88.7±1.0 | | 29.4±0.2 | | 34.8±3.5 | |  |
| 15 | 110±5 | 7.0±1.0 | 2201±229 | | 316±13 | 91.7±1.9 | | 30.0±0.3 | | 60.5±6.4 | |  |
| 16 | 125±3 | 5.5±1.4 | 1348±211 | | 255±19 | 93.5±0.9 | | 31.1±0.3 | | 39.1±5.8 | |  |
|  |  |  |  |  | |  |  | |  | |  | |

**Table S6** Agronomic traits of hybrid rice F_1_s

| Combinations | | GD | PH(cm) | PN | NG | NGP | SF(%) | GW(g) | | YP(g) |  |
| --- | --- | --- | --- | --- | --- | --- | --- | --- | --- | --- | --- |
| JH2A/9311 | 115±2.5^fgh^ | | 124±2^d^ | 7.3±0.6^b^ | 1608±166^d^ | 219±10^de^ | 75.9±3.6^abcd^ | | 25.4±0.2^de^ | 31.0±4.1^de^ | |
| JH2A/SH527 | 117±1.5^f^ | | 123±3^d^ | 8.3±1.5^ab^ | 1793±472^bcd^ | 213±19^ef^ | 51.6±7.0^e^ | | 25.3±0.7^de^ | 22.8±2.6^f^ | |
| JH2A/R345 | 115±0.6f^gh^ | | 124±3^d^ | 7.7±1.2^b^ | 2146±337^ab^ | 280±18^a^ | 78.1±3.7^abcd^ | | 24.7±0.6^e^ | 41.1±3.4^abc^ | |
| JH2A/R346 | 114±0.0^h^ | | 125±1^d^ | 7.7±1.2^b^ | 2050±176^ab^ | 269±21^ab^ | 78.2±2.3^abc^ | | 24.7±0.3^e^ | 39.6±2.9^bc^ | |
| JH2A/R348 | 114±0.6^gh^ | | 124±2^d^ | 8.3±0.6^ab^ | 2319±87^a^ | 279±9^a^ | 75.0±4.1^abcd^ | | 25.7±0.2^d^ | 44.5±1.0^a^ | |
| LFA/9311 | 121±1.2^e^ | | 126±3^cd^ | 7.0±1.0^b^ | 1660±129^cd^ | 239±16^cd^ | 0^f^ | | - | - | |
| LFA/SH527 | 114±1.2^gh^ | | 122±3^d^ | 8.3±1.2^ab^ | 1548±150^d^ | 187±15^g^ | 72.8±1.7^cd^ | | 25.4±0.2^de^ | 28.6±3.2^e^ | |
| LFA/R345 | 116±1.0^fg^ | | 123±5^d^ | 8.3±0.6^ab^ | 2386±69^a^ | 287±11^a^ | 73.5±2.4^bcd^ | | 25.2±0.5^de^ | 44.1±1.8^ab^ | |
| LFA/R346 | 115±0.6^fgh^ | | 123±2^d^ | 7.3±0.6^b^ | 2025±65^abc^ | 277±12^ab^ | 76.4±2.3^abcd^ | | 25.3±0.9^de^ | 39.1±1.3^c^ | |
| LFA/R348 | 115±1.5^fgh^ | | 124±4^d^ | 8.3±1.2^ab^ | 2234±278^a^ | 269±24^ab^ | 75.0±4.6^abcd^ | | 25.9±0.7^cd^ | 43.1±4.0^abc^ | |
| GZ4S/9311 | 138±0.6^a^ | | 140±5^a^ | 8.3±0.6^ab^ | 1585±54^d^ | 191±11^fg^ | 76.0±2.3^abcd^ | | 27.5±0.4^ab^ | 33.1±1.7^de^ | |
| GZ4S/SH527 | 134±1.2^b^ | | 135±2^ab^ | 9.7±0.6^a^ | 1627±54^d^ | 169±10^g^ | 78.5±3.2^ab^ | | 26.7±0.6^bc^ | 34.1±2.1^d^ | |
| GZ4S/R345 | 129±1.2^d^ | | 138±2^a^ | 7.7±1.2^b^ | 2073±195^ab^ | 272±14^ab^ | 79.5±0.8^a^ | | 25.8±0.3^cd^ | 42.6±3.8^abc^ | |
| GZ4S/R346 | 131±0.6^c^ | | 136±3^ab^ | 8.0±0.0^ab^ | 2023±67^abc^ | 253±8^bc^ | 72.5±1.5^d^ | | 27.7±0.6^a^ | 40.6±2.5^abc^ | |
| GZ4S/R348 | 130±0.6^cd^ | | 132±4^bc^ | 8.0±1.7^ab^ | 2152±383^ab^ | 271±17^ab^ | 78.0±4.7^abcd^ | | 25.5±0.8^de^ | 42.4±4.0^abc^ | |

Note: JH2A, Honglian-CMS line Jiahong-2A; LFA, WA-CMS line Luofei-A; GZ4S, two-line photo-thermo-sensitive genic male sterility line Guangzhan4S. Letters indicated a significant difference at the 5% significance level by the least significant difference test. “-” indicated no investigation results.
